# Impact of Cardiovascular Risk Factors on Medical Expenditure: Evidence From Epidemiological Studies Analysing Data on Health Checkups and Medical Insurance

**DOI:** 10.2188/jea.JE20140096

**Published:** 2014-11-05

**Authors:** Koshi Nakamura

**Affiliations:** Department of Epidemiology and Public Health, Kanazawa Medical University, Uchinada, Ishikawa, Japan; 金沢医科大学公衆衛生学

**Keywords:** cardiovascular risk factor, medical expenditure, cohort study

## Abstract

Concerns have increasingly been raised about the medical economic burden in Japan, of which approximately 20% is attributable to cardiovascular disease, including coronary heart disease and stroke. Because the management of risk factors is essential for the prevention of cardiovascular disease, it is important to understand the relationship between cardiovascular risk factors and medical expenditure in the Japanese population. However, only a few Japanese epidemiological studies analysing data on health checkups and medical insurance have provided evidence on this topic. Patients with cardiovascular risk factors, including obesity, hypertension, and diabetes, may incur medical expenditures through treatment of the risk factors themselves and through procedures for associated diseases that usually require hospitalization and sometimes result in death. Untreated risk factors may cause medical expenditure surges, mainly due to long-term hospitalization, more often than risk factors preventively treated by medication. On an individual patient level, medical expenditures increase with the number of concomitant cardiovascular risk factors. For single risk factors, personal medical expenditure may increase with the severity of that factor. However, on a population level, the medical economic burden attributable to cardiovascular risk factors results largely from a single, particularly prevalent risk factor, especially from mildly-to-moderately abnormal levels of the factor. Therefore, cardiovascular risk factors require management on the basis of both a cost-effective strategy of treating high-risk patients and a population strategy for reducing both the ill health and medical economic burdens that result from cardiovascular disease.

## Abstract (ja)

日本では医療費財源の負担が懸念されているが、その約20%は冠動脈疾患と脳卒中を合わせた循環器疾患に起因するものである。循環器疾患の予防には危険因子の管理が必須であるため、日本人集団における循環器疾患危険因子と医療費の関係を理解することが重要である。しかし、健康診断と医療保険のデータを解析して、この話題に関するエビデンスを提供した日本の疫学研究は数少ない。肥満、高血圧、糖尿病などの循環器疾患危険因子は、それ自身に対する治療と入院を要して死に至ることもある関連疾病に対する治療を通じて医療費を発生させるかもしれない。危険因子に対して治療が行われている場合と比べて、未治療の場合は主として長期入院による医療費の急騰が起こる可能性がさらに高いかもしれない。個人レベルでは、保有する危険因子の数に伴って医療費が増える。単一の危険因子に着目すれば、その重症度に伴って医療費が増えるかもしれない。しかし、集団レベルでは、循環器疾患危険因子に起因する医療費財源の負担の多くは頻度の多い単一の危険因子、特にその軽度～中等度の異常から生じているものである。このため、循環器疾患による健康障害および医療費財源の負担の両方を軽減させるためには、費用対効果を考慮したハイリスクアプローチと集団アプローチの両方にもとづいた危険因子の管理が必要である。

## INTRODUCTION

In Japan, the majority of outpatient medical services and hospitalizations are provided at approved clinics and hospitals within the medical insurance system, which regulates medical costs nationwide and is compulsory for all Japanese residents.^1^^–^^3^ Thus, people spend public resources to receive medical services, even though they pay up to 30% of medical expenditures for medical services they receive, in addition to monthly insurance fees. As this medical economic burden is increasing drastically each year,^1^ concerns regarding this issue have been raised.

Cardiovascular disease, including coronary heart disease and stroke, accounts for 20.8% of the total medical expenditure in the Japanese population and is the leading cause of medical economic burden.^1^ Because the management of risk factors is essential for the prevention of cardiovascular disease, it is important to understand the relationship between cardiovascular risk factors and medical expenditure in the Japanese population. However, only a few Japanese epidemiological studies analysing data on health checkups and medical insurance have provided evidence on this topic.

## AN INDIVIDUAL PERSPECTIVE ON THE RELATIONSHIP BETWEEN CARDIOVASCULAR RISK FACTORS AND MEDICAL EXPENDITURE

The Shiga National Health Insurance (NHI) cohort study was one of the earliest Japanese studies on this topic.^4^^–^^12^ It was conducted in 7 towns and 1 village in Shiga prefecture in the central part of Japan and included 4535 community dwellers between the ages of 40 and 69 years who were beneficiaries of NHI, an insurance group for self-employed individuals (eg farmers and fishermen) as well as retirees and their dependants. The participants were followed up for 10 years following their health checkup in 1989–1991 in order to calculate the monthly medical expenditure during the follow-up period and to identify deceased cases. If a participant withdrew or died, the follow-up period was terminated at that time. The study obtained information on medical expenditures per participant and information on cases that withdrew from the NHI system or died, using the monthly claim history from the Shiga NHI organizations. The medical expenditure recorded in this study was restricted to the fee schedule range used in the medical insurance system in Japan, calculated as the sum of the expenditures from the insurance organization and from the beneficiary.

The arithmetic mean medical expenditure was calculated for participants grouped according to baseline characteristics measured at the health checkup. When subtracting the arithmetic mean medical expenditure of participants without the risk factor of interest from the corresponding mean of those with the factor, this residual medical expenditure crudely represented the average medical expenditure attributable to the risk factor of interest. Data on medical expenditure was logarithmically transformed to normalize the positively skewed medical expenditure distribution. Analysis of covariance was then used to compare medical expenditure strictly among characteristic groups after adjustment for potential confounding factors, including age, sex, and other cardiovascular risk factors. Log-transformed values were ultimately expressed as geometric mean medical expenditure. The present study hypothesized that a patient with a cardiovascular risk factor incurred medical expenditures not only through treatment of the factor itself but also through very expensive procedures required for associated severe vascular diseases. To support this hypothesis, the hazard ratio for all-cause mortality was also calculated using a Cox proportional hazards model incorporating the same covariates.

**Figure 1.**
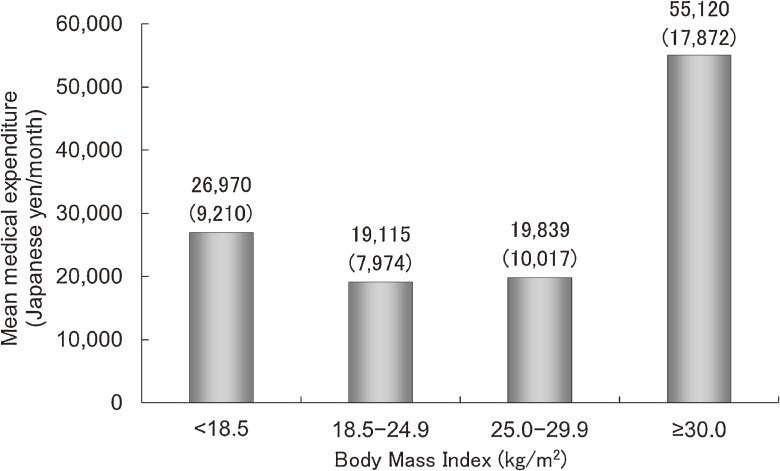
The crude arithmetic mean medical expenditure per month over 10 years of follow-up in Japanese medical insurance beneficiaries 40–69 years of age, grouped according to their body mass index. Numbers in parentheses represent the geometric means after adjustment for age, sex, and smoking and drinking habits (analysis of covariance, *P* < 0.01).^9^ Note: data edited for currency translation.

According to the obesity classifications of the World Health Organization^13^ and the Japan Society for the Study of Obesity,^14^ the participants were categorized into one of the following 4 groups: body mass index (BMI) <18.5 kg/m^2^; BMI 18.5–24.9 kg/m^2^; BMI 25.0–29.9 kg/m^2^; and BMI ≥30.0 kg/m^2^. A J-shaped relationship existed between BMI and medical expenditure, with the nadir of the curve occurring at a BMI of 18.5–24.9 kg/m^2^, even after adjustment for potential confounders (Figure 1).^9^ In contrast, BMI showed an inversely J-shaped relationship with all-cause mortality. Underweight participants may have incurred medical expenditures and increased mortality risks as a result of a pre-existing severe disease that potentially contributed to their low weight at baseline.^15^

**Figure 2.**
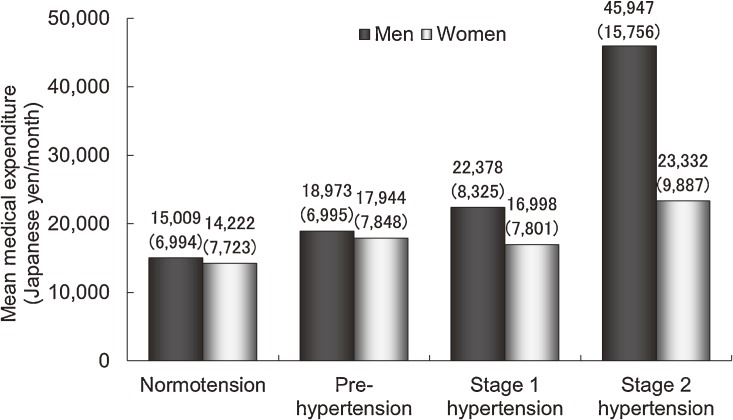
The crude arithmetic mean medical expenditure per month over 10 years of follow-up in male and female Japanese medical insurance beneficiaries 40–69 years of age, grouped according to their sex and hypertension status. Numbers in parentheses represent the geometric means after adjustment for age, body mass index, smoking and drinking habits, serum total cholesterol levels, and a history of diabetes (analysis of covariance, *P* < 0.01 for men and *P* = 0.18 for women).^5^

According to the 7th report of the Joint National Committee in the United States,^16^ the participants who were not taking antihypertensive medication and did not have a history of cardiovascular disease were categorized into one of the following 4 groups: normotension (systolic blood pressure [SBP] <120 mm Hg and diastolic blood pressure [DBP] <80 mm Hg); pre-hypertension (SBP 120–139 mm Hg and/or DBP 80–89 mm Hg); stage 1 hypertension (SBP 140–159 mm Hg and/or DBP 90–99 mm Hg); and stage 2 hypertension (SBP ≥160 mm Hg and/or DBP ≥100 mm Hg). Medical expenditure tended to increase with the severity of hypertension, especially in men (Figure 2).^5^ The risk of all-cause mortality was also higher in stage 2 hypertensive men than in normotensive men, with a multivariate-adjusted hazard ratio of 3.19 (95% confidence interval [CI] 1.67–6.08).

Medical expenditure was higher in diabetic participants, who were defined as having a history of diabetes as assessed by a self-reported questionnaire, than in non-diabetic participants (crude arithmetic mean 39 479 vs. 19 573 Japanese yen/month; multivariate-adjusted geometric mean 15 788 vs. 8325 Japanese yen/month; *P* < 0.01).^11^ The risk of all-cause mortality tended to be higher in diabetic than in non-diabetic participants, with a multivariate-adjusted hazard ratio of 1.45 (95% CI 0.86–2.47).

Medical expenditure was higher in participants who had proteinuria, identified using a spot urine dipstick test, than in those who did not have this condition (crude arithmetic mean 37 494 vs. 20 029 Japanese yen/month; multivariate-adjusted geometric mean 14 200 vs. 8451 Japanese yen/month; *P* < 0.01).^8^ Proteinuria tended to increase the risk of all-cause mortality, with a multivariate-adjusted hazard ratio of 1.60 (95% CI 0.64–4.03).

**Figure 3.**
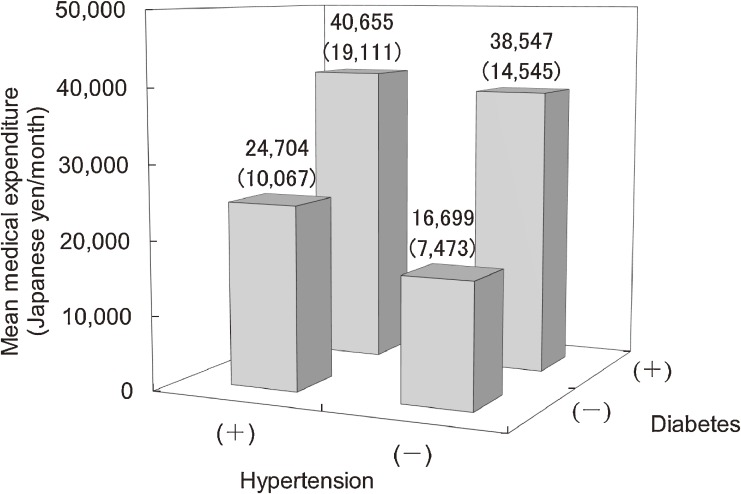
The crude arithmetic mean medical expenditure per month over 10 years of follow-up in Japanese medical insurance beneficiaries 40–69 years of age, grouped according to their hypertension and diabetes statuses. Numbers in parentheses represent geometric mean after adjustment for age, sex, body mass index, smoking drinking habits, and serum total cholesterol levels (analysis of covariance, *P* < 0.01).^7^

Hypertension and diabetes coexist in a single individual mainly due to insulin resistance accompanied by compensatory hyperinsulinemia,^17^^,^^18^ which occurs more frequently in obese than in non-obese individuals.^19^^,^^20^ The participants were categorized into one of the following 4 groups: neither hypertension nor diabetes; hypertension alone; diabetes alone; or both hypertension and diabetes. Hypertension was defined as SBP ≥140 mm Hg, DBP ≥90 mm Hg, and/or taking antihypertensive medication, while diabetes was defined as described above. Participants with both hypertension and diabetes had the highest medical expenditure among the 4 groups (Figure 3).^7^ These participants also had the highest risk of all-cause mortality, with a multivariate-adjusted hazard ratio of 2.21 (95% CI 1.11–4.42), relative to those with neither hypertension nor diabetes.

Medical expenditure broadly increased with the number of concomitant cardiovascular risk factors, including hypertension, diabetes, and hypercholesterolemia, which was defined as a serum total cholesterol ≥220 mg/dL.^10^ Although this pattern was similar in participants both with and without obesity, defined as a BMI ≥25.0 kg/m^2^, obesity resulted in additional medical expenditures related to hypertension, diabetes, and hypercholesterolemia in each group.

## A POPULATION PERSPECTIVE ON THE RELATIONSHIP BETWEEN CARDIOVASCULAR RISK FACTORS AND MEDICAL EXPENDITURE

The Shiga NHI cohort study estimated medical expenditures attributable to cardiovascular risk factors from a population perspective using the population attributable fraction concept as follows:

$$\begin{array}{l} ([\mathrm{arithmetic mean of medical expenditure in participants}\\ \;\mathrm{who had a risk factor of interest}-\mathrm{arithmetic mean of}\\ \;\mathrm{medical expenditures in participants who did not have}\\ \;\mathrm{the factor}]\times \mathrm{number of participants who had the factor})\\ \;\mathrm{/medical expenditure incurred in the entire population} \end{array}$$

This represents the possible reduction in medical expenditure, given that the risk factor of interest is eliminated from the entire population completely.

The medical expenditure attributable to obesity (BMI ≥25 kg/m^2^) accounted for 3.1% of that incurred in the entire population.^9^

**Figure 4.**
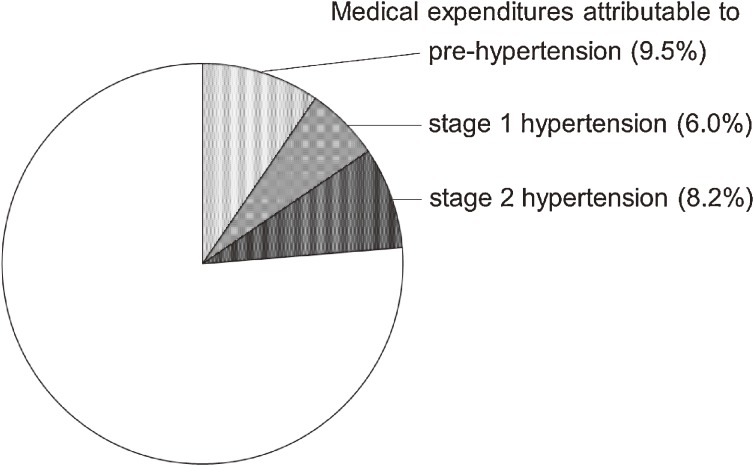
The percentage of medical expenditures attributable to pre-, stage 1, and stage 2 hypertension for the entire population of Japanese medical insurance beneficiaries 40–69 years of age (19 090 468 Japanese yen/month/1000 persons, 100%).^5^

The medical expenditure attributable to hypertension (including pre-hypertension) accounted for 23.7% of that incurred in the entire population (Figure 4).^5^ The medical expenditure percentages according to the hypertension grade were 9.5% for the pre-hypertension group, 6.0% for the stage 1 hypertension group, and 8.2% for the stage 2 hypertension group.

The medical expenditure attributable to obesity and/or metabolic disorders, including hypertension, diabetes, and hypercholesterolemia, accounted for 23.6% of that incurred in the entire population.^10^ The percentages were 13.1% for the presence of any 1 of the 3 metabolic disorder without obesity, 3.4% for the presence of any 2 or 3 of the 3 metabolic disorders without obesity, 2.4% for obesity alone, 1.8% for the presence of any 1 of the 3 metabolic disorders plus obesity, and 2.9% for the presence of any 2 or 3 of the 3 metabolic disorders plus obesity.

## EVIDENCE DERIVED FROM RELEVANT LARGE COHORT STUDIES

The second Shiga NHI cohort study included NHI beneficiaries from all 26 local municipalities of Shiga prefecture and collected data from their health checkup in 2000 and medical expenditures for up to 6 years between the fiscal years of 2000 and 2005.^21^

The mean medical expenditure per year during the follow-up period was calculated for participants 40 years of age or older (*n* = 33 213) who were grouped by age and sex according to the number of cardiovascular risk factors each participant had at baseline: none of 4 cardiovascular risk factors (hypertension, hypercholesterolemia, high blood glucose, and current smoking); any 1 of the 4 factors; any 2 of the 4 factors; and any 3 or 4 of the 4 factors. These 4 cardiovascular risk factors were defined as follows: hypertension, defined as SBP ≥140 mm Hg and/or DBP ≥90 mm Hg; hypercholesterolemia, defined as serum total cholesterol ≥240 mg/dL; high blood glucose, defined as casual blood glucose ≥200 mg/dL; and smoking, defined as currently smoking. The study estimated the adjusted mean medical expenditure on a real scale for the 4 age- and sex-specific groups using a gamma regression model, which is a type of generalised linear model.^22^ This model has recently been considered the best modelling approach to handle medical expenditure data that allows for potential confounders. The mean medical expenditure increased with the number of concomitant cardiovascular risk factors, irrespective of age or sex.^21^ The mean medical expenditure was 6-to-7-fold higher for participants at age 80 who had any 3 or 4 of the 4 risk factors (men: 603 351 Japanese yen/year; women: 765 673 Japanese yen/year) compared with those at age 50 who had none of the 4 risk factors (men: 110 708 Japanese yen/year; women: 107 109 Japanese yen/year).

A tremendously large cohort study collected similar data throughout Japan and included beneficiaries of both NHI (12 local organizations) and the Employee’s Health Insurance scheme (9 local organizations).^23^ Currently, approximately two-thirds of all Japanese people younger than 75 years of age are enrolled in Employee’s Health Insurance schemes available to employees and their dependants, while the remaining are enrolled in NHI.^1^ The study collected data on medical expenditure for the year of 2009 after their health checkup in 2008.

**Figure 5.**
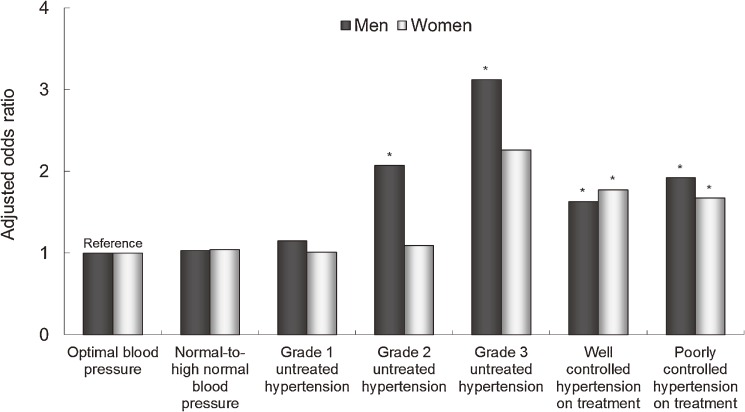
The adjusted odds ratios for being in the top 1% medical expenditure group over 1 year of follow-up in male and female Japanese medical insurance beneficiaries 40–54 years of age, grouped according to their hypertension status. Adjustments were made for age, body mass index, smoking habits, serum low-density lipoprotein cholesterol levels, log-transformed fasting plasma glucose levels, and medications for hypercholesterolemia and diabetes (a logistic regression model, *P < 0.05 vs. optimal blood pressure).^23^

Moturu et al suggested that a very small portion of patients accounted for a substantial percentage of the medical expenditure in the entire population.^24^ In fact, male and female study participants who ranked in the top 1% for medical expenditures incurred costs of at least 1 807 710 and 1 437 650 Japanese yen/year, respectively (data edited for currency translation), while the respective medical expenditure sums in these top 1% male and female groups accounted for 25.6% and 21.2% of medical expenditures incurred in the entire male and female populations 40–69 years of age (*n* = 314 622).^23^ The corresponding male and female groups showed median cumulative hospitalization periods of 38 and 32 days, respectively. The study compared the risk of being in the top 1% medical expenditure group in the year following the baseline survey among age- and sex-specific participants grouped according to blood pressure and antihypertensive medication status at baseline. Participants who were not taking antihypertensive medication were categorized into 1 of the following 5 groups defined according to the 2007 criteria of the European Society of Hypertension and the European Society of Cardiology^25^: optimal blood pressure (SBP <120 mm Hg and DBP <80 mm Hg); normal-to-high-normal blood pressure (SBP 120–139 mm Hg and/or DBP 80–89 mm Hg); grade 1 hypertension (SBP 140–159 mm Hg and/or DBP 90–99 mm Hg); grade 2 hypertension (SBP 160–179 mm Hg and/or DBP 100–109 mm Hg); and grade 3 hypertension (SBP ≥180 mm Hg and/or DBP ≥110 mm Hg). The remaining participants, who were taking antihypertensive medication, were classified into one of the following 2 groups: well-controlled hypertension on treatment (SBP <140 mm Hg and DBP <90 mm Hg on medication); and poorly-controlled hypertension on treatment (SBP ≥140 mm Hg and/or DBP ≥90 mm Hg on medication). The risk of incurring such extremely high medical expenditures increased with more severe, untreated hypertension in men aged 40–54 or 55–69 years and in women aged 40–54 years (Figure 5; results presented only for men and women aged 40–54 years). In men and women aged 40–54 years, the grade 2-to-3 untreated hypertension group had an even greater risk of incurring higher medical expenditures than the well-controlled hypertension on treatment group.

## CONCLUSIONS AND FUTURE DIRECTIONS

Patients with cardiovascular risk factors may incur medical expenditures though both treatments of the risk factors themselves and procedures for associated diseases that usually require hospitalization and sometimes result in death. Untreated risk factors may cause medical expenditure surges, mainly due to long-term hospitalization, more often than risk factors preventatively treated by medication. On an individual patient level, medical expenditures increase with the number of concomitant risk factors. For single risk factors, personal medical expenditure may increase with the severity of that factor. However, on a population level, the medical economic burden attributable to cardiovascular risk factors results largely from a single, particularly prevalent risk factor, especially from mildly-to-moderately abnormal levels of the factor. This is explained by Rose’s theory that ‘a large number of people exposed to a small risk may generate many more cases than a small number exposed to high risk’.^26^ Therefore, cardiovascular risk factors require management on the basis of 2 strategies for reducing both the ill health and medical economic burdens resulting from cardiovascular disease. One is a cost-effective strategy of treating high-risk individuals based on the assumption that the treatment of cardiovascular risk factors themselves is essential for preventing cardiovascular disease in individuals with risk factors. The second is a population strategy which aims to shift the entire population to more desirable physical profiles from current ones. Risk factors that are directly linked to undesirable lifestyle habits, such as smoking and excessive alcohol intake, might be important targets for this purpose, as lifestyle modifications may lead directly to cost-effective elimination of the corresponding risk factors and subsequently to reduction of the overall medical economic burden.^12^^,^^27^

To reduce both the ill health and medical economic burdens resulting from cardiovascular disease, the Japanese national government introduced a nationwide public health strategy in 2008.^1^^,^^28^^,^^29^ Individuals with cardiovascular risk factors, including mildly elevated blood pressure and mildly deregulated lipid and glucose metabolism, especially those with obesity, are considered candidates for incurring medical expenditure; consequently, various modes of intervention are used in these individuals, depending on the number and severity of risk factors. Therefore, medical staff and health care planners need to acknowledge the importance of this issue. Larger cohort studies are expected to provide more reliable and useful evidence on this subject. It is also important to contribute new statistical analysis methods and interesting ideas to this topic in future studies.

## ONLINE ONLY MATERIALS

Abstract in Japanese.
