# Supplementary material for: Impact of Cardiovascular Risk Factors on Medical Expenditure: Evidence From Epidemiological Studies Analysing Data on Health Checkups and Medical Insurance
Source: J Epidemiol. 2014 Nov 5;24(6):437–43. doi: 10.2188/jea.JE20140096 (PMC4213217; doi:10.2188/jea.JE20140096)
Supplement: Abstract in Japanese. [file je-24-437-s001.pdf]

循環器疾患危険因子が医療費に及ぼす影響：健康診断と医療保険のデータを解析した疫学研究からのエビデンス

金沢医科大学公衆衛生学
中村幸志

日本では医療費財源の負担が懸念されているが、その約 20%は冠動脈疾患と脳卒中を合わせた循環器疾患に起因するものである。循環器疾患の予防には危険因子の管理が必須であるため、日本人集団における循環器疾患危険因子と医療費の関係を理解することが重要である。しかし、健康診断と医療保険のデータを解析して、この話題に関するエビデンスを提供した日本の疫学研究は数少ない。肥満、高血圧、糖尿病などの循環器疾患危険因子は、それ自身に対する治療と入院を要して死に至ることもある関連疾病に対する治療を通じて医療費を発生させるかもしれない。危険因子に対して治療が行われている場合と比べて、未治療の場合は主として長期入院による医療費の急騰が起こる可能性がさらに高いかもしれない。個人レベルでは、保有する危険因子の数に伴って医療費が増える。単一の危険因子に着目すれば、その重症度に伴って医療費が増えるかもしれない。しかし、集団レベルでは、循環器疾患危険因子に起因する医療費財源の負担の多くは頻度の多い単一の危険因子、特にその軽度～中等度の異常から生じているものである。このため、循環器疾患による健康障害および医療費財源の負担の両方を軽減させるためには、費用対効果を考慮したハイリスクアプローチと集団アプローチの両方にもとづいた危険因子の管理が必要である。

キーワード
循環器疾患危険因子、医療費、コホート研究
